# Fabrication of Drug-Eluting Polycaprolactone/poly(lactic-*co*-glycolic Acid) Prolapse Mats Using Solution-Extrusion 3D Printing and Coaxial Electrospinning Techniques

**DOI:** 10.3390/polym13142295

**Published:** 2021-07-13

**Authors:** Yi-Pin Chen, Tsia-Shu Lo, Yu-Ting Lin, Yu-Han Chien, Chia-Jung Lu, Shih-Jung Liu

**Affiliations:** 1 Department of Obstetrics and Gynecology, Keelung Chang Gung Memorial Hospital, Keelung 20401, Taiwan; cmjackchen@hotmail.com; 2 Department of Obstetrics and Gynecology, Chang Gung Memorial Hospital-Linkou, School of Medicine, Chang Gung University, Taoyuan 33305, Taiwan; 2378@cgmh.org.tw; 3 Department of Mechanical Engineering, Chang Gung University, Taoyuan 33302, Taiwan; yutinna9876@mail.cgu.edu.tw (Y.-T.L.); luhan871202@gmail.com (Y.-H.C.); happy2231017@mail.cgu.edu.tw (C.-J.L.); 4 Bone and Joint Research Center, Department of Orthopedic Surgery, Chang Gung Memorial Hospital-Linkou, Taoyuan 33305, Taiwan

**Keywords:** prolapse membrane, solution-extrusion 3D printing, coaxial electrospinning, polycaprolactone, poly(lactic-*co*-glycolic acid), nanofibers

## Abstract

We developed biodegradable drug-eluting prolapse mats using solution-extrusion 3D printing and coaxial electrospinning techniques. The mats were composed of polycaprolactone (PCL) mesh and lidocaine-, estradiol-, metronidazole-, and connective tissue growth factor (CTGF)-incorporated poly(lactic-*co*-glycolic acid) (PLGA) nanofibers that mimic the structure of the natural extracellular matrix of most connective tissues. The mechanical properties of degradable prolapse membrane were assessed and compared to commercial non-degradable polypropylene knitted meshes clinically used for pelvic organ prolapse (POP) repair. The release behaviors of the drug-loaded hybrid degradable membranes were also characterized. The experimental results suggest that 3D-printed PCL meshes exhibited comparable strengths to commercial POP meshes and survived through 10,000 cycles of fatigue test without breakage. Hybrid PCL meshes/PLGA nanofibrous membranes provided a sustainable release of metronidazole, lidocaine, and estradiol for 4, 25, and 30 days, respectively, in vitro. The membranes further liberated high levels of CTGF for more than 30 days. The animal tests show that the mechanical property of PCL mesh decreased with time, mainly due to degradation of the polymers post-implantation. No adverse effect of the mesh/nanofibers was noted in the histological images. By adopting solution-extrusion 3D printing and coaxial electrospinning, degradable drug-eluting membranes can be fabricated for POP applications.

## 1. Introduction

Female pelvic organ prolapse (POP) arises when organs, such as the bladder, uterus, or rectum, swag and compress the vagina [1,2,3]. POP is a major public health issue with a high rate of incidence in the aging populations in developed countries. Synthetic meshes made of polypropylene (PP) materials have been adopted to provide post-surgical pelvic assist and avoid peri-operative complications correlated with autologous fascia harvesting. Mesh exposure, mesh erosion, and infection have been reported as primary complications originating from the employment of synthetic graft/mesh, with an incidence of 7–25% [4]. Contemporaneous vaginal hysterectomy is related to an enhanced risk of vaginal mesh erosion. Aggregated anterior/posterior vaginal mesh therapy is further a raised hazardous factor for intraoperative bleeding and blood transfusion. Knowledge of synthetic polymeric meshes utilized for incontinence surgery confirms the benefit of monofilament PP mesh when compared to multifilament PP mesh. However, investigations on the microstructure of the meshes intimate that long-time tissue reaction may be inferior to that of biological grafts [5,6].

An ideal mesh for POP repair should fulfill the following criteria: (1) have the adequate mechanical strength to support the pelvic floor; (2) possess favorable flexibility to assist implantation and fixation; (3) convey appropriate drug/growth factor concentrations to the target site for pain relief and infection control, as well as the formation of connective tissues; (4) be resorbable after fulfilling its function and be biocompatible such that the material degradation procedure would not lead to any tissue irritation.

In this study, we developed hybrid degradable mesh/drug-eluting nanofibrous membranes for the repair of POP. Polycaprolactone (PCL) mesh was fabricated using a lab-developed solution-extrusion 3D printer [7], while estradiol-, lidocaine-, metronidazole-, and connective tissue growth factor (CTGF)-loaded poly(lactic-*co*-glycolic acid) (PLGA) core-shell nanofibers were prepared by employing electrospinning and coaxial electrospinning techniques [8,9].

3D printing has been regarded as the newest technology that achieves novel innovations and solves complicated medical issues in pharmaceutical and medical research. This adaptable technology has completed tangible and innovative advances because of the increasing demand for customized equipment/devices for personalized treatment and diagnostics [10]. Coaxial electrospinning, on the other hand, is an effective and prompt technique for manufacturing sheath-core-structured nanofibrous membranes [11]. Electrospun membranes with biomolecules entrapped at the core can provide tailorable and sustainable release of drugs. Additionally, the membranes possess high surface area and three-dimensional nanofibrous networks allowing the electrospun fibers to imitate native extracellular matrices. These characteristics enable the nanofibrous membranes great potential in terms of applications for drug delivery and tissue engineering.

PCL is a semi-crystalline polymer possessing a melting temperature of 59~64 °C and a glass transition temperature of −60 °C. Due to its non-toxicity and biocompatibility, PCL is widely employed as resorbable sutures, scaffolds in regenerative therapy, and carriers in drug delivery systems. PCL displays an extended degradation time (longer than 2 years) and is resorbed via microorganisms or hydrolysis of the aliphatic ester linkage under physiological conditions [12]. In contrast, PLGA is one of the most widely used polymeric biomaterials in controlled drug deliveries, not only due to its biocompatibility, biodegradability, and favorable release kinetics, but also its capability in protein/biomolecule delivery. PLGA has also been utilized to deliver various small-molecule drugs, peptides, and proteins, including fertility regulating hormones, growth hormones, steroid hormones, anti-inflammatory drugs, cytokines, chemotherapeutics, antibiotics, narcotic antagonists, insulin, and vaccines [13].

Lidocaine is a local anesthetic agent that is widely employed as a regional and topical anesthetic, an antiarrhythmic and analgesic, as well as an adjunct to tracheal intubation [14]. Estradiol, an estrogen steroid hormone, is the primary female sex hormone that is involved in the management of the estrous and menstrual female reproductive cycles. Estradiol is essential in the evolution and retention of female reproductive tissues, including the mammary glands, uterus, and vagina, during puberty, adulthood, and pregnancy. The pelvic organs and their surrounding muscular and connective tissue support are estrogen-responsive [15]. Metronidazole is an antibiotic widely used in different medical conditions, such as trichomoniasis, amebiasis, and giardiasis, among others. The use of metronidazole has been extended to the therapy of nonspecific vaginitis and anaerobic infections [16].

The mechanical properties of 3D-printed meshes were determined by a tensile tester. The structure of the drug-loaded sheath-core nanofibers was evaluated using scanning electron microscopy (SEM), transmission electron microscopy (TEM), and laser scanning confocal microscopy (LSCM). The release profiles of pharmaceuticals/biomolecules from the nanofibers were also assessed utilizing high-performance liquid chromatography (HPLC) and enzyme-linked immunosorbent assay (ELISA).

## 2. Materials and Method

### 2.1. 3D-Printed Degradable Meshes

**Figure 1:**
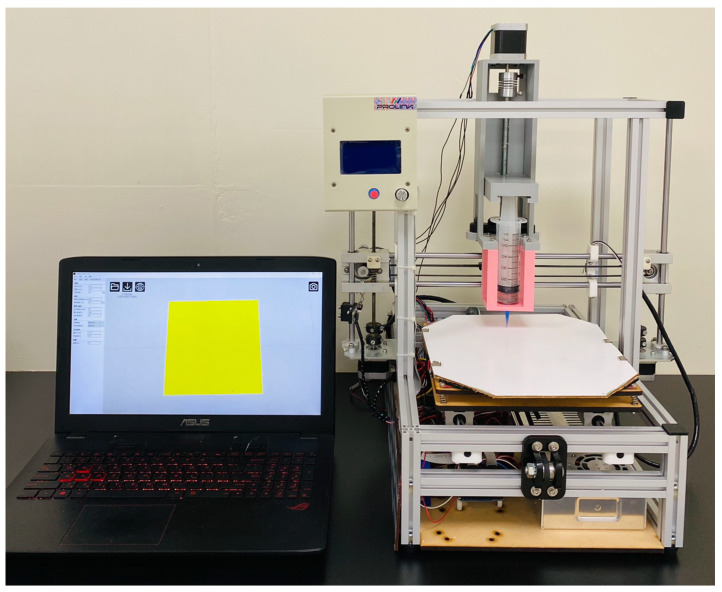
Photo of the lab-made solution-extrusion 3D printer.

Polycaprolactone (PCL) with a molecular weight of 80,000 Da was used as the mesh material, and dichloromethane (DCM) was employed as the solvent; both were purchased from Sigma-Aldrich (St. Louis, MO, USA). Degradable meshes were manufactured utilizing a lab-developed extrusion 3D printer (Figure 1) [7]. PCL (2500 mg) were stir blended with DCM (3.5 mL) for 2 h. The mixture was then charged into the extrusion setup of the printer, which is equipped with a syringe and a delivering nozzle for printing. In the printing procedure, the nozzle was steered by a computer-commanded motor, and the PCL mixture was extruded on the collection table. Once the solvent was vaporized, PCL meshes with a pore size of 3 mm and a thickness of 0.5 mm were obtained on the collection table.

### 2.2. Drug/Biomolecule-Loaded Sheath-Core-Structured Nanofibrous Membranes

50:50 PLGA with a molecular weight of 33,000 Da (Sigma-Aldrich, St. Louis, MO, USA) was used as the nanofiber material, while 1,1,1,3,3,3-hexafluoro-2-propanol (HFIP) (Sigma-Aldrich) was used as the solvent. Human recombinant connective tissue growth factor (CTGF) was employed as the biomolecule.

Bi-layered drugs/biomolecules-loaded nanofibrous membranes, which consist of an ordinary layer and a sheath/core-structured layer, were fabricated. A few test trials were first completed to identify the compositions of polymers and drugs that can successfully fabricate the nanofibrous membranes. For manufacturing the ordinary nanofiber layer, PLGA (896 mg), metronidazole (112 mg), and estradiol (112 mg) were primarily mixed with 4 mL of HFIP and subsequently electrospun via a lab-made electrospinning device. The delivery speed of the mixture was 0.7 mL/h.

After the electrospinning of the ordinary layer, the sheath/core nanofibrous layer was spun. For the sheath-core nanofibrous layer, PLGA (896 mg) and lidocaine (224 mg) were dissolved in 4 mL of HFIP as the sheath material, while the core mixture consisted of 1 mL of CTGF (20 μg) mixed with 1 mL of phosphate-buffered saline (PBS). A dedicated co-axial electrospinning apparatus that concurrently conveys two mixtures to a collection sheet was employed to spin the nanofibers [17]. The sheath-layer PLGA/lidocaine and the core CTGF mixtures were charged into two separate feeding syringes. In the co-spinning process, the mixtures were spun and transported to the collecting plate at volumetric flow rates of 0.24 mL/h for the shell PLGA/lidocaine mixture and 0.08 mL/h for the core CTGF mixture via two distinct pumps. Electrospinning tests were performed at 25 °C. Hybrid bi-layered nanofibrous membranes were thus obtained. The thickness of the bi-layered membrane acquired was approximately 0.2 mm (each layer was approximately 0.1 mm). All spun membranes were deposed in an isothermal oven at 40 °C for 3 days to vaporize the solvent and thereafter stored at 4 °C until usage.

### 2.3. Mechanical Properties

The mechanical properties of 3D-printed PCL meshes were estimated utilizing tensile test equipment (Lloyd, Ametek, Berwyn, PA, USA) with a 2.5 kN load cell. The maximum strengths of two commercially PP meshes currently used in POP repair, namely HAlbaMesH and Surelift meshes, were also assessed for comparisons. Specimens (2 cm × 5 cm) were cut from the 3D-printed PCL and commercial PP meshes for the tests. The stretching speed was set at 100 mm/min and the ultimate load and deformation were recorded.

### 2.4. Molecular Weight Variations

The molecular weight variation of the 3D-printed PCL meshes was characterized by immersing the meshes in a phosphate buffer at 37 °C. The samples were taken out of the buffered solution at various weeks. After drying, the molecular weights were evaluated by a gel permeation chromatograph (GPC) equipped with a Waters 2414 refractive index detector (Waters Corp, Milford, MA, USA).

### 2.5. Fatigue Test

The fatigue properties of PCL mesh were characterized to determine the ability of the meshes to withstand conditions of cyclic fatigue loading. Tests of PCL meshes were evaluated on the tensiometer using a cyclic loading mode. The meshes were stretched by 20% of their initial lengths and then returned to zero loadings for 10,000 cycles. The total number of cycles before fracture for each mesh was recorded. Both the PCL and commercial PP meshes were evaluated. Three fatigue tests (*n* = 3) were completed for each mesh.

### 2.6. Scanning Electron Microscope (SEM) Assessment

The morphological structure of electrospun nanofibrous membranes was assessed using SEM, and the size distribution of nanofibers was estimated from 100 arbitrarily chosen fibers (*n* = 3) employing a commercial ImageJ code (National Institutes of Health, Bethesda, MD, USA).

### 2.7. Transmission Electron Microscope (TEM) Observation

The sheath/core structure of nanofibers was confirmed using a JEOL Model JEM-2000EXII TEM (Tokyo, Japan).

### 2.8. Confocal Microscopy

The existence of active biomolecules in the co-spun sheath/core nanofibers was verified. Nanofibers with PLGA as the sheath and reGFP (6H1-38, Shanghai PrimeGene Bio-Tech, China) as the core were electrospun and examined under a laser scanning confocal microscope (LSCM) (Leica TS SP8X, Tokyo, Japan). The wavelength for reGFP observation was set at 487 nm.

### 2.9. Contact Angle of Water

To examine the hydrophilicity, water contact angles of pure PLGA, metronidazole/estradiol-embedded nanofibers, and lidocaine/CTGF-embedded sheath-core nanofibers were measured. Water was mildly dropped onto the surface of nanofibrous membranes (1 cm × 1 cm) and analyzed using a video monitor (*n* = 3).

### 2.10. Fourier Transform Infrared Spectroscopy (FTIR)

To obtain the infrared spectrum of the drug-loaded mats, FTIR was performed on the Bruker Tensor 27 spectrometer at a resolution of 4 cm^−1^ in the absorption mode, with a total of 32 scans. The membrane sample was pressed into KBr discs, and the absorption spectrum was recorded in the range of 400–4000 cm^−1^.

### 2.11. In Vitro Release of Drugs/Growth Factors

The release profiles of metronidazole, estradiol, lidocaine, and CTGF from the drug-embedded biodegradable nanofibers were examined using an in vitro elution scheme. Membrane samples (approximately 20 mm × 30 mm, 200 mg) were deposed in assay tubes (*n* = 3), each containing 1 mL of PBS. The tubes were incubated at 37 °C for 24 h before the mixture was gathered and assayed. Fresh PBS (1 mL) was filled into the tubes for the following 24-h incubation, and the process was continued for 30 days.

The pharmaceutical levels in the gathered mixtures were assessed using HPLC, which was performed on a Hitachi L-2200R multi-solvent delivery system (Tokyo, Japan). Acetonitrile:PBS at a ratio of 30:70 (*v*/*v*) (pH 7.0) was used as the mobile phase for metronidazole [18]. The wavelength and flow rate for the assay were 319 nm and 1.0 mL/min, respectively. A Symmetry C8 (5 µm, 4.6 mm × 250 mm) column (Supelco, Sigma-Aldrich, St. Louis, MO, USA) was used for the assay. The retention time was set at 3.067 min. In contrast, acetonitrile:DI water at a ratio of 45:55 (*v*/*v*) was used as the mobile phase for estradiol [19]. Absorbance was recorded at a wavelength of 281 nm, while the flow rate was 1.0 mL/min. The retention time was set at 9.167 min. Acetonitrile:heptan at a ratio of 25:75 (*v*/*v*) was used as the mobile phase for lidocaine [20]. Absorbance was recorded at a wavelength of 205 nm, while the flow rate was set at 1.0 mL/min. A Symmetry C8 (5 µm, 4.6 mm × 150 mm) column (Waters Corp, Milford, MA, USA) was used for the assay. The retention time was set at 10.240 min. Meanwhile, the CTGF levels were measured using ELISA. Eluents of 100 μL were added to the appropriate microtiter plate wells with a biotin-conjugated antibody and incubated for 90 min at 37 °C. Avidin conjugated to Horseradish Peroxidase (HRP) was then added to each microplate well and incubated for 30 min. After the addition of sulphuric acid solution, the color variation was measured spectrophotometrically at 450 nm. The concentration of CTGF in the eluents was quantified by comparing the optical density of the specimens to the standard curve. All experiments were performed in triplicate (*n* = 3).

### 2.12. In Vivo Animal Test

**Figure 2:**
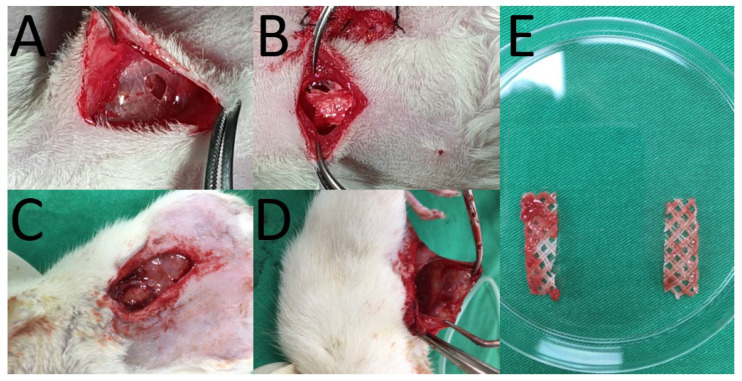
Polycaprolactone (PCL) meshes implantation. (**A**) expose the peritoneum (**B**) put the PCL mesh on peritoneum. Retrieve the PCL meshes at 28 days post-implantation. (**C**) Expose the mesh, (**D**) cut mesh from peritoneum, (**E**) retrieved meshes.

Ten Sprague-Dawley rats (about 300 g) were treated and cared for under the supervision of a licensed veterinarian, in a manner consistent with the regulations of the National Institute of Health of Taiwan. All animal related procedures were approved by the Institutional Animal Care and Use Committee of Chang Gung Memorial Hospital (IACUC Approval No.: CGMH2018121905). As shown in Figure 2, the rats were first anesthetized via isoflurane, and a 4-cm incision was placed in the lower abdomen of each animal. After the implantation of PCL mesh/nanofibrous membranes (30 mm × 10 mm × 0.5 cm in size), the wound was closed with 3-0 Vicryl sutures. Five animals were sacrificed at 30 days. The mesh/nanofibrous membranes were retrieved and their tensile properties were characterized. Another five animals were sacrificed for local tissue sampling at post-operative days 1, 4, 7, and 28 days for histological examination.

## 3. Results

### 3.1. Tensile Properties of Printed PCL Mesh

**Figure 3:**
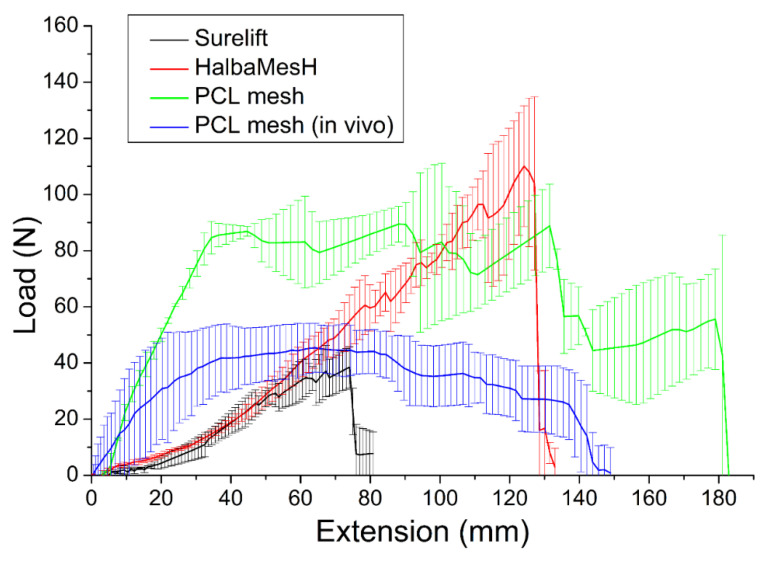
Tensile properties of 3D-printed polycaprolactone (PCL) meshes, retrieved PCL meshes in the in vivo experiments, and commercially available Surelift and HalbaMesH prolapse meshes.

Figure 3 illustrates the tensile curves of printed PCL meshes. The PCL meshes (97.1 ± 19.2 N) exhibited inferior strength to that of HAlbaMesH PP meshes (129.7 ± 18.4 N), yet superior to that of Surelift meshes (42.3 ± 8.9 N), demonstrating the potential of PCL meshes as an assisting scaffold for pelvic floor repair.

**Figure 4:**
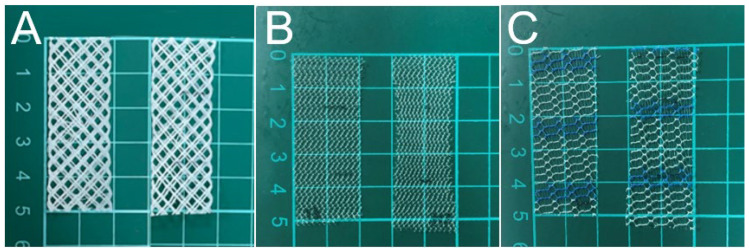
(**A**) 3D-printed polycaprolactone (PCL), (**B**) Surelift and (**C**) HalbaMesH prolapse meshes after 10,000 cycles of fatigue tests.

The fatigue properties of PCL and commercial PP meshes was assessed by subjecting them to cyclic extension by 20% of their length for 10,000 cycles. All meshes survived through 10,000 cycles without breakage, as shown in Figure 4. However, the meshes tended to elongate after the fatigue test. The extended part lengths for the 3D-printed PCL, HAlbaMesH, and Surelift meshes were 2.0%, 11.5%, and 9.2%, respectively. PCL mesh showed smaller residual strains than the commercial PP meshes, suggesting that the degradable mesh can be a good candidate as scaffold for pelvic floor repair.

Meanwhile, the GPC analysis results in Table 1 show that the molecular weights of printed meshes decrease with time, indicting the ongoing degradation of PCL.

### 3.2. Characterization of Electrospun Nanofibers

**Figure 5:**
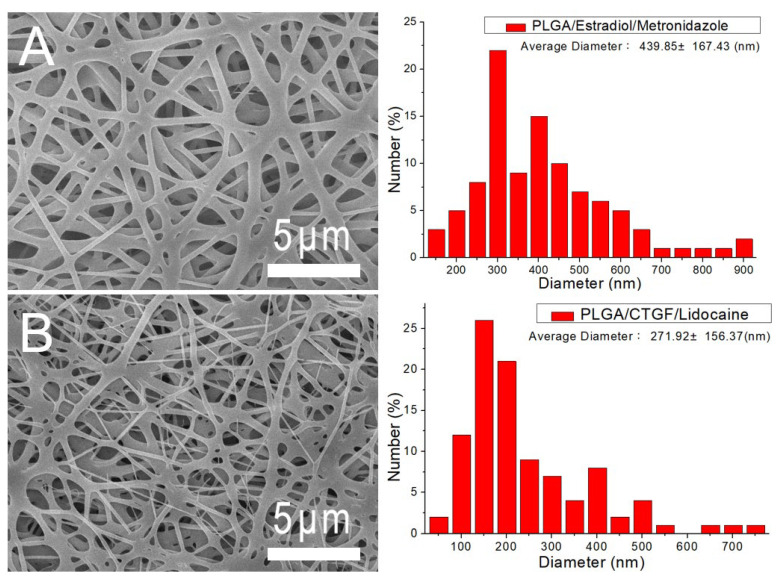
Scanning electron microscopy (SEM) images and fiber diameter distribution. (**A**) Drug-loaded poly (lactic-co-glycolic acid) (PLGA) nanofibers and (**B**) sheath-core-structured PLGA/connective tissue growth factor (CTGF) nanofibers.

Drugs/biomolecules-loaded nanofibrous membranes were manufactured employing suitable process parameters. Figure 5A,B displays the structures of the metronidazole- and estradiol-loaded PLGA nanofibers and sheath-core-structured PLGA/lidocaine/CTGF nanofibers, respectively, at a magnification of 6000×. The sizes of electrospun metronidazole/estradiol/PLGA nanofibers (439.8 ± 167.4 nm) were greater than those of the lidocaine/CTGF/PLGA nanofibers (271.9 ± 156.4 nm). In the electrospinning procedure, the polymeric mixture is stretched by the external electric force. Compared to the regular nanofibers, the sheath-core-structured nanofibers do not contain polymer material at the core. Therefore, the nanofibers can be easily extended by the exterior force. The sizes of the electrospun fibers decreased accordingly.

**Figure 6:**
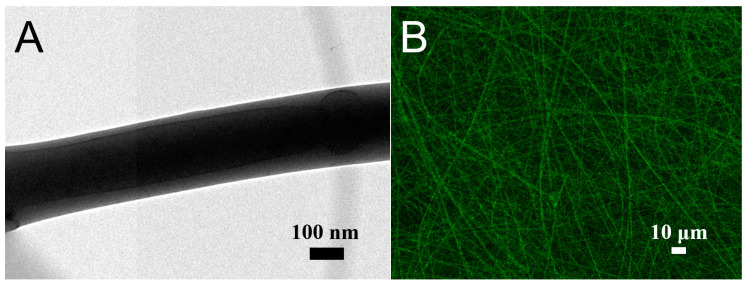
(**A**) Transmission electron microscopy (TEM) image, and (**B**) laser scanning confocal microscope image of sheath-core nanofibers.

Figure 6 displays the TEM and LSCM images. The liquid solution could be identified (darker colored) in the PLGA/lidocaine/CTGF nanofibers (Figure 6A), exhibiting a sheath-core structure. The LSCM image in Figure 6B shows thread-like reGFP in sheath-core-structured nanofibers. All these demonstrated the successful fabrication of sheath-core-structured nanofibers by the co-axial electrospinning technique.

**Figure 7:**
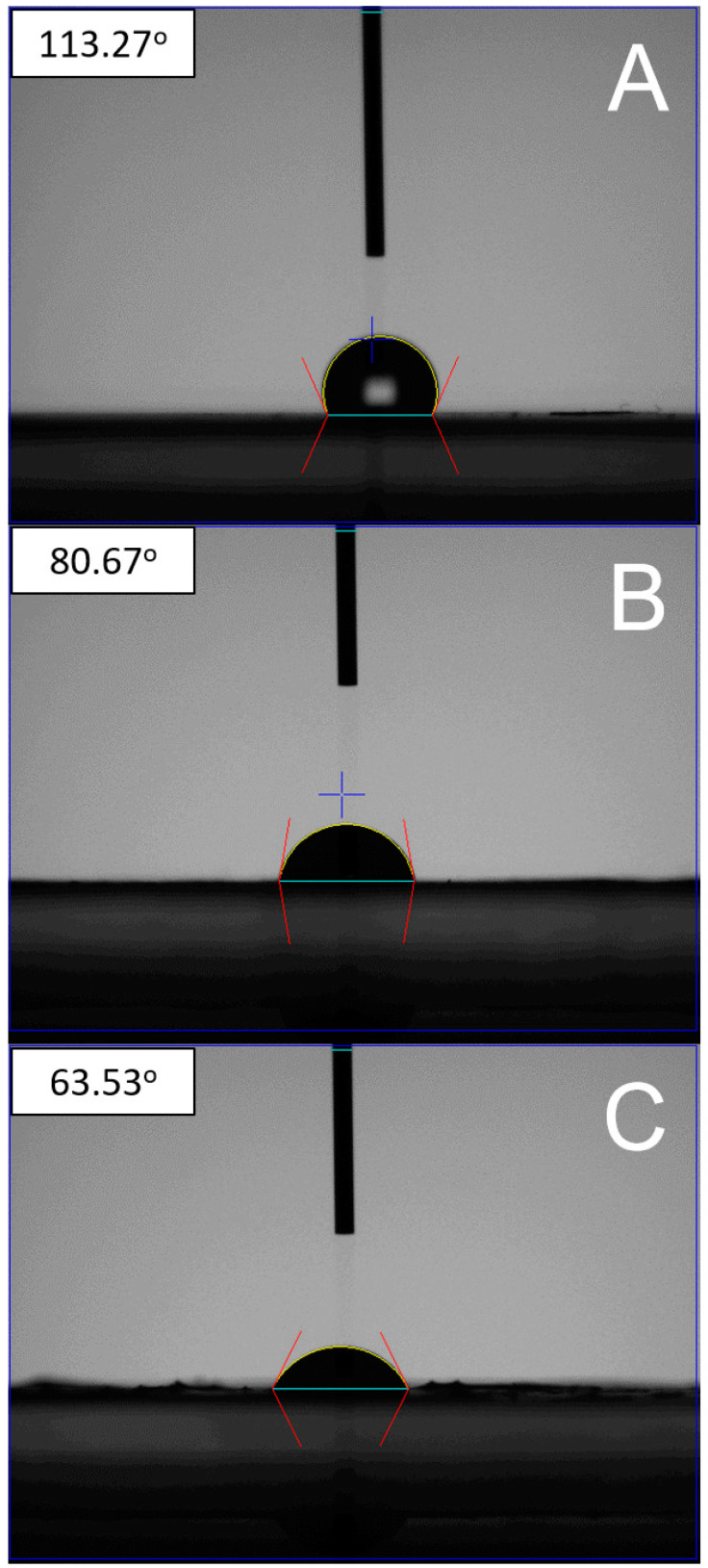
Water contact angles of (**A**) pure poly (lactic-*co*-glycolic acid) (PLGA), 113.3°, (**B**) drug-loaded PLGA, 80.6°, (**C**) sheath-core connective tissue growth factor (CTGF)-embedded PLGA nanofibers, 63.5°.

Figure 7 presents the measured contact angles of pure PLGA, PLGA/metronidazole/estradiol, and PLGA/lidocaine/CTGF membranes. The estimated contact angle for pure PLGA nanofibers was 113.3°, while the water angles were 80.6° and 63.5° for the PLGA/metronidazole/estradiol and PLGA/lidocaine/CTGF nanofibers, respectively. While the pure PLGA nanofibers showed hydrophobic features, the presence of water-soluble pharmaceuticals such as metronidazole and lidocaine greatly enhanced the hydrophilicity of the spun nanofibers.

**Figure 8:**
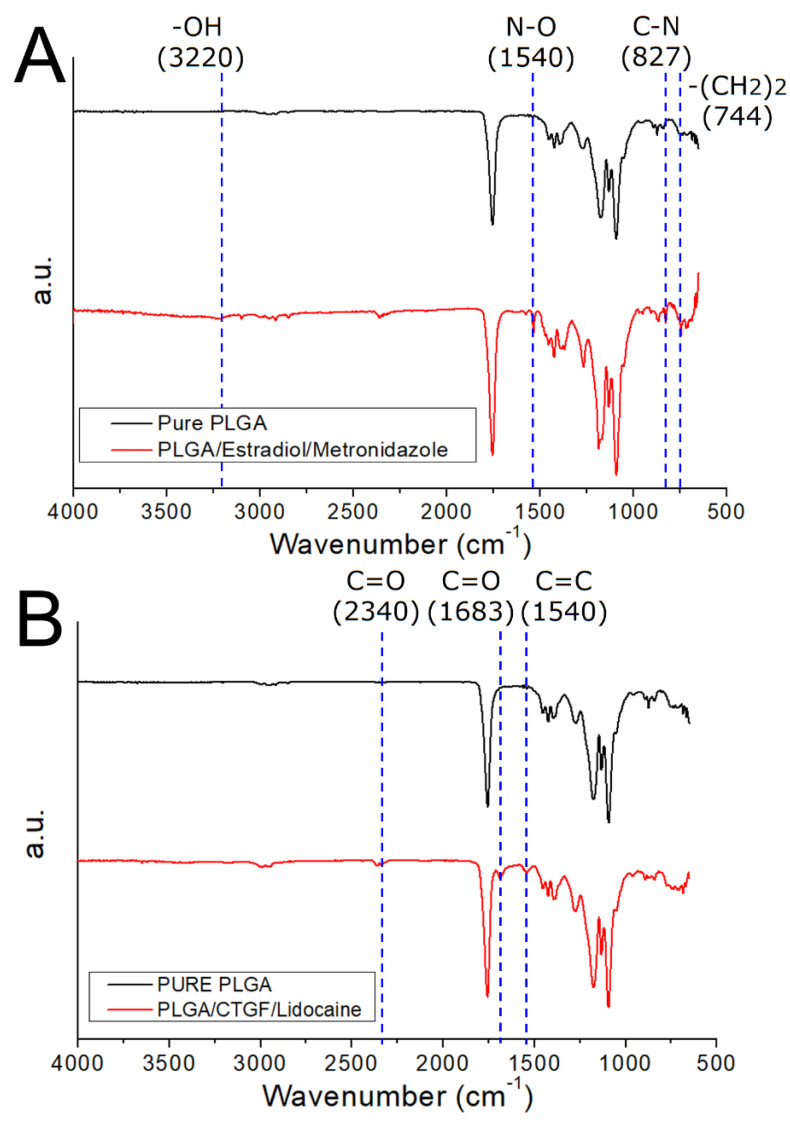
Fourier-transform infrared spectroscopy (FTIR) spectra, (**A**) drug-loaded poly (lactic-co-glycolic acid) (PLGA) nanofibers, (**B**) connective tissue growth factor (CTGF)-embedded sheath-core nanofibers.

Figure 8A displays the FTIR assay of pure PLGA membranes and metronidazole/estradiol-incorporated PLGA membranes. The peaks at 3220 and 744 cm^−1^ were enhanced due to the –OH and –(CH_2_)_2_ bonds of added biomolecules, respectively [21]. The new vibration peaks at 1540 cm^−1^ and 827 cm^−1^ were mainly owing to the N–O and C–N bonds of the metronidazole [22]. In contrast, Figure 8B displays the spectra of pure PLGA- and lidocaine/CTGF-embedded nanofibers. The vibration peaks at 2340 and 1683 cm^−1^ were enhanced owing to the C=O bond of lidocaine, while the new peak at 1540 cm^−1^ resulted from the C=C of the analgesics [23]. The FTIR spectra assay confirmed that the pharmaceuticals have been successfully embedded in the PLGA membranes.

### 3.3. In Vitro Elution

**Figure 9:**
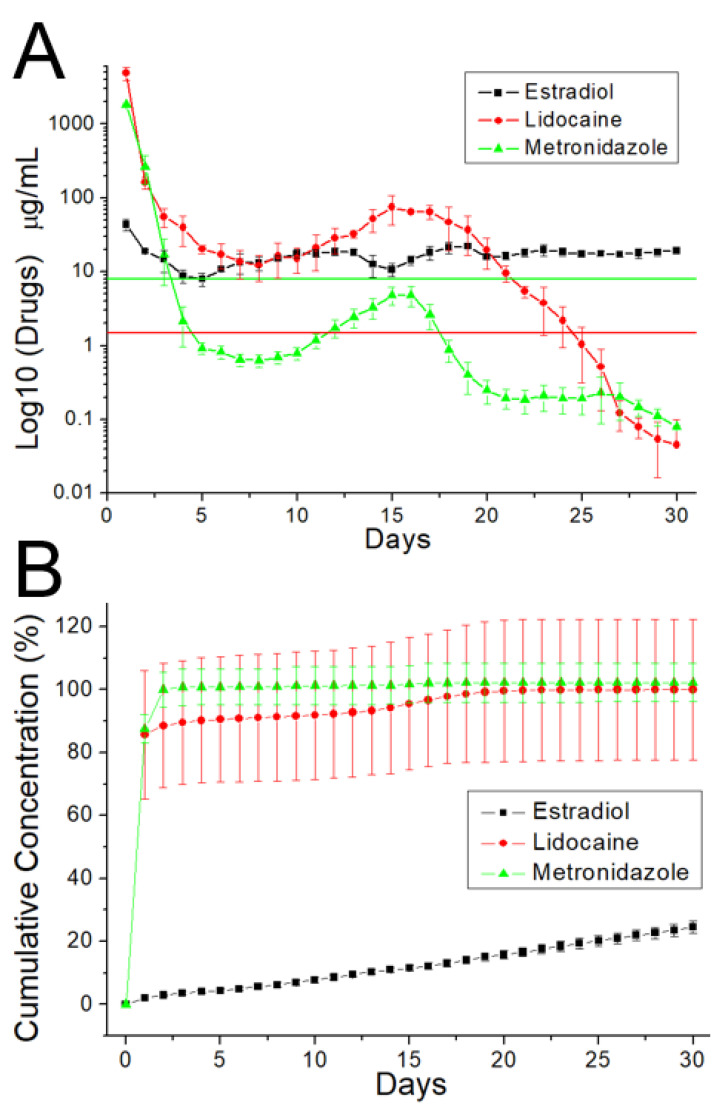
In vitro (**A**) daily, (**B**) cumulative release of pharmaceuticals from the nanofibers. The minimum inhibitory concentrations (MICs) of metronidazole and lidocaine were 8 and 1.4 μg/mL. Meanwhile, the minimum therapeutic concentration (MTC) of estradiol was 1.36 pg/mL, which is too low to be shown in the upper figure.

**Figure 10:**
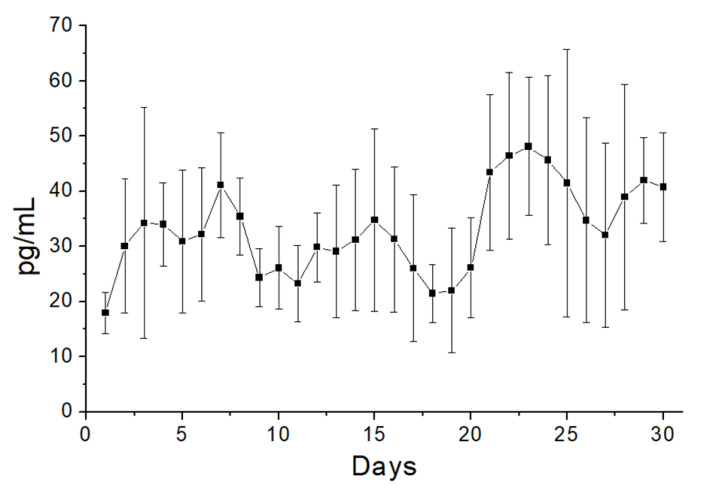
In vitro of connective tissue growth factors (CTGFs) from the sheath-core nanofibers.

Figure 9 illustrates the daily and cumulative liberation profiles of metronidazole, estradiol, and lidocaine from the nanofibers in vitro. Both metronidazole and lidocaine exhibited a tri-phase liberation feature, i.e., a burst at day 1, a second peak release near 15 days, followed by a steady and gradually diminishing drug elution. The release of estradiol showed a tiny burst and more steady elution through the entire study period. Additionally, the electrospun nanofibrous membranes sustainably liberated effective metronidazole (above the minimum inhibitory concentration) [24] and lidocaine (above the minimum therapeutic concentration) for 4 and 21 days, respectively [25,26]. Meanwhile, the membranes also provided sustained release of estradiol for over 30 days. In contrast, the ELISA data revealed that the PLGA nanofibers sustainably eluted CTGF for over 30 days (Figure 10).

### 3.4. In Vivo Results

Figure 2E illustrates the retrieved PCL meshes. All meshes appeared to be intact after implantation in animals for 30 days. The mechanical strengths of retrieved meshes were assessed and the result is shown in Figure 3. The experimental outcomes suggest that the maximum of PCL mesh decreased from 89.4 ± 6.3 to 45.4 ± 8.7 N after implanted in rats for 28 days, demonstrating degradation of the polymeric material with time and matching with the in vitro results in Table 1. Despite the strength reduction, the retrieved mesh still exhibited superior mechanical property to the commercially available HAlbaMesH meshes.

**Figure 11:**
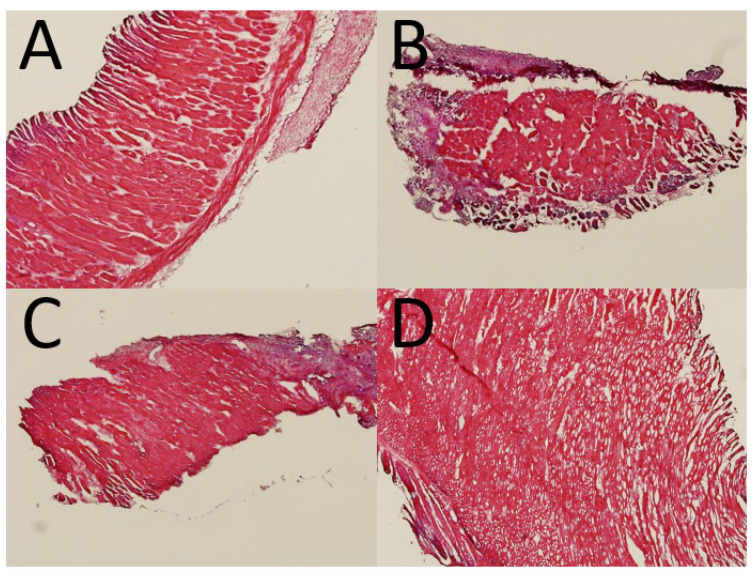
Histological images at (**A**) 1, (**B**) 4, (**C**) 7, and (**D**) 28 days post implantation.

Figure 11 illustrates the hematoxylin & eosin (H&E) images of the mesh/nanofibrous membranes. The histological images at post-operative days 1, 4, 7, 14 and 28 revealed no inflammation increased leukocytes or tissue necrosis in all groups. That is, no adverse effect was noted in the histological images.

## 4. Discussion

Non-absorbable meshes have been widely used to reinforce the pelvic floor for recurrent pelvic organ prolapse patients after native tissue repair (NTR) surgery [27]. However, mesh erosion occurs and induces complications, such as tissue infection, etc., after mesh implantation surgery. Some patients have to receive another surgery to remove the non-absorbable meshes [28].

In this study, we developed biodegradable hybrid PCL mesh/drug-eluting PLGA nanofibrous membranes for the repair of POP, using 3D printing and electrospinning techniques. 3D printing provides the advantages of freedom of design, mass customization, waste minimization, the capability to manufacture complex parts, as well as fast prototyping [29]. The 3D-printed PCL meshes exhibited superior strengths to those of commercial PP membranes. Furthermore, the meshes survived through 10,000 cyclic loadings [30] and showed the least residual strains. These properties provide advantages in terms of their long-term use for POP repair.

Local delivery of pharmaceuticals offers high and sustainable drug concentrations at the target tissue with minimum systemic doses and related possibility of hypoglycemia risk. Electrospun PLGA fibers have been widely researched for tissue engineering applications owing to their unique feature that intimates the micro-/nano-fibrous morphology of the extracellular matrix. Ideal nanofibers should have high porosity, a high aspect ratio, and great strengths to achieve enhanced tissue proliferation. Mukherjee et al. [31] proposed that nanofibrous mesh supplies a distinct topography that allows entrapment of therapeutic cells for up to six weeks, thereby enhancing substantial cellular infiltration of host anti-inflammatory macrophages. Implementing appropriate cell attachment to the scaffold is the key to success. Nevertheless, there remains a challenge in that employing highly hydrophobic scaffold mats may lead to less-than-optimal cell colonization. The experimental results suggested that the addition of pharmaceuticals into the nanofibrous POP mats enhances their hydrophilicity, which in turn enhances the proliferation of connective tissues for POP repair [32]. In addition, the drugs and CTGF-trapped nanofibrous PLGA membranes demonstrated excellent flexibility and extensibility, thus promoting their applications in pelvic floor repair that allows for tissue contraction during healing.

In general, drug release from a drug-embedded resorbable device consists of three distinct stages, including burst release, diffusion-governed elution, and degradation-governed discharge. During electrospinning, a majority of loaded drugs are encapsulated in the volume of the polymeric matrix. Nonetheless, some pharmaceuticals might be located on surfaces of the nanofibrous membranes, therefore resulting in the burst release. After the burst, the drug-liberation feature is controlled by both diffusion and polymer degradation. A second peak drug discharge was thus observed for lidocaine and metronidazole; thereafter, the release profile diminished gradually. Relatively, the nanofibers eluted estradiol smoothly. The experimental data affirmed that the electrospun drug-embedded membranes discharge high concentrations of lidocaine and estradiol for 21 and more than 30 days, respectively, which is beneficial in terms of pain control [20,33] as well as helps reduce vaginal symptoms of menopause (such as vaginal dryness/burning/itching) [34]. Nevertheless, the nanofibers released an effective level (higher than the minimum inhibitory concentrations (MICs)) for only 4 days. Tugzu-Demiroz et al. [35] prepared metronidazole-loaded nanofibers via electrospinning, and proposed that metronidazole-loaded nanofibers can be a potential drug delivery system for the treatment of bacterial vaginosis. Srithep et al. [36] developed metronidazole-loaded polylactide stereocomplex electrospun nanofiber mats for treatment of periodontal disease. They found that the entrapment efficiency of metronidazole in the nanofiber mats was 82–99% and at least 30% of metronidazole was released in the initial period followed by a sustained-release for up to 7 days. To further prolong the sustained release of loaded pharmaceuticals in the PLGA mats, a higher drug loading, a greater polymer-to-drug ratio, a polymer of higher molecular weight [37], and/or a highly aligned fiber structure [38] may be adopted. On the other hand, owing to the shielding influence of the shell PLGA layer, the initial burst of CTGF from the sheath-core-structured nanofibers was not observed. However, a steady discharge was observed for more than 30 days. The result implies that CTGF-incorporated PLGA nanofibrous membranes can be used as an efficacious scaffold to promote connective tissue generation for POP repair.

Limitations existed in this preliminary work. This study employed the rat model to test the drugs/molecules loaded nanofibers. The relevance of our findings to humans with pelvic organ prolapse remains unclear and needs to be further explored. These will be the topics of our future studies.

## 5. Conclusions

This study exploited degradable hybrid PCL mesh/drug-eluting PLGA nanofibers that mimic the morphology of the natural extracellular matrix of most connective tissues, using solution-extrusion 3D printing and coaxial electrospinning techniques. The mechanical properties of degradable meshes were assessed and compared to those of commercial non-degradable PP knitted macroporous ultra-lightweight implants, clinically employed for POP repair. The drug release behaviors of the mesh were also characterized. The experimental results suggest that the meshes could sustainably release effective levels of estradiol, lidocaine, and metronidazole for 30, 25, and 4 days, respectively, in vitro. Meanwhile, the mesh also released high concentrations of connective tissue growth factor for over 30 days. Therefore, 3D-printed mesh/multi-drug-loaded nanofibrous membranes may provide advantages in terms of reduced postoperative complications as well as improved POP therapies.

## Figures and Tables

**Table 1:** Molecular weight variations of biodegradable meshes with time.

Week	*M*n	*M*w	*M*z	*M*z + 1
0	73,430	87,747	120,764	196,001
2	70,694	83,577	113,851	184,945
4	65,064	73,174	87,902	117,305
8	64,851	72,824	86,631	113,841

*M*n: number average molecular weight, *M*w: weight average molecular weight, *M*z: z average molecular weight, *M*z + 1: z + 1 average molecular weight.

## Data Availability

The data used to support the findings of this study are included within the article.
